# Physical and Morphological Changes of Poly(tetrafluoroethylene) after Using Non-Thermal Plasma-Treatments

**DOI:** 10.3390/ma11102013

**Published:** 2018-10-17

**Authors:** Jorge López-García, Florence Cupessala, Petr Humpolíček, Marian Lehocký

**Affiliations:** 1 Centre of Polymer Systems, University Institute, Tomas Bata University in Zlín, Trida Tomase Bati 5678, 76001 Zlín, Czech Republic; humpolicek@utb.cz (P.H.); lehocky@utb.cz (M.L.); 2 École supérieure de Chimie Organique et Minérale ESCOM, Allée du Réseau Jean Marie Buckmaster, 60200 Compiègne, France; florence.cupessala05@gmail.com

**Keywords:** Poly(tetrafluoroethylene), Teflon, plasma treatment, zeta potential, surface energy, contact angle measurement

## Abstract

A commercial formulation of poly(tetrafluoroethylene) (PTFE) sheets were surface modified by using non-thermal air at 40 kHz frequency (DC) and 13.56 MHz radiofrequency (RF) at different durations and powers. In order to assess possible changes of PTFE surface properties, zeta potential (ζ), isoelectric points (IEPs) determinations, contact angle measurements as well as Scanning Electron Microscopy (SEM) and Atomic Force Microscopy (AFM) imaging were carried out throughout the experimentation. The overall outcome indicated that ζ-potential and surface energy progressively changed after each treatment, the IEP shifting to lower pH values and the implicit differences, which are produced after each distinct treatment, giving new surface topographies and chemistry. The present approach might serve as a feasible and promising method to alter the surface properties of poly(tetrafluoroethylene).

## 1. Introduction

**Figure 1:**
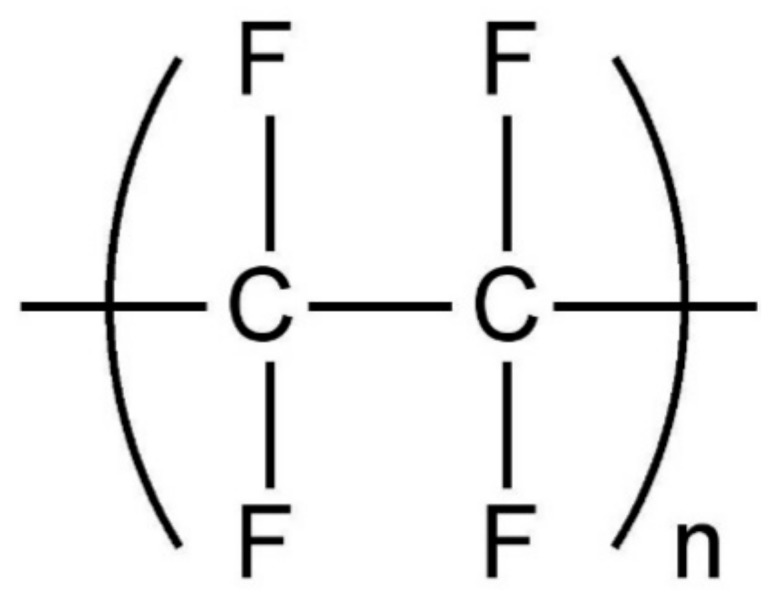
Chemical structure of poly(tetrafluoroethylene) (PTFE).

One straightforward strategy to modify certain surface properties without altering polymer bulk properties is by using non-thermal plasma technologies, such as corona, dielectric barrier, radiofrequency and microwave discharges. Furthermore, plasma treatment is a very versatile technique, since various carrier gases may be employed, giving unique features to the treated material [1,2,3,4,5]. Poly(tetrafluoroethylene) (PTFE), known commercially as Teflon^®^ is a type of fluorinated polymer formed by a succession of molecules of two fluorine atoms (F) and one of carbon (C), its chemical structure is similar to polyethylene; instead of having carbon and hydrogen atoms, the latter are replaced by fluorine atoms as shown in Figure 1.

The strong cohesive force between fluorine and carbon makes poly(tetrafluoroethylene) an inert and nonstick material. Except for other PTFE-like molecules, there is no other molecule that adheres to it [6,7,8]. One of PTFE’s properties is thermal stability, since it is one of the most thermostable plastic materials; for instance it does not show any decomposition above 250 °C, keeping most of its properties. PTFE’s thermal conductivity coefficient does not change with temperature, and is relatively high, and thus it is a good insulator. The mixture of PTFE with other materials like glass fibers or carbon increases its thermal conductivity and its resistance to chemical agents. Regarding resistance to atmospheric agents and light, it has been shown that PTFE specimens do not drastically change their properties after being exposed to extreme environment conditions. This inert material reacts only with fluorinated hydrocarbons and fluorinated oils at high temperatures (above 300 °C) causing some swelling and dissolution, which may be reversible. The molecular configuration of PTFE gives the surface a high anti-adhesion. Hence, PTFE surfaces are unwettable and are deemed as a super hydrophobic material [9,10,11,12,13]. PTFE withstands elevated temperatures, and unlike typical thermoplastics, its viscosity above the melting point is so high that PTFE may not be processed by traditional methods, such as extrusion or injection molding. For this reason, PTFE components are manufactured by means of special compression molding and sintering techniques to create blocks, sheets and rods. Recently though, some modified PTFE materials may already be processed as thermoplastics by using traditional techniques as well as electrospinning, which is one of the simplest ways to produce the polymer fibers with nano-sized porous media that has a high surface area per unit volume [14,15,16,17].

The aim of this contribution was to make a comprehensive assessment of the effect of air plasma treatment on PTFE commercial sheets by using 40 kHz (DC) and 13.56 MHz (RF) plasma discharges at different plasma duration and power inputs. This outcome is supported by surface probe techniques, such as Scanning Electron Microscopy (SEM), Atomic Force Microscopy (AFM), zeta potential analysis and contact angle measurements. The extensive and indispensable use of PTFE in our daily life underlies the motivation of choosing this material as the target of this work.

## 2. Materials and Methods

### 2.1. Materials

Poly(tetrafluoroethylene) (PTFE) was obtained from Dow Chemical Company, (Midland, MI, USA). Distilled-deionized water was retreated in a Simplicity UV^®^ unit, Milipore S.A.S, Molsheim, France, equipped with dual wavelength 185/254 nm UV lamp. The ultra-pure water was used for all experiments and solutions with a water resistivity of 18.2 MΩ at 25 °C and total organic carbon content lower than 5 ppb. Potassium chloride KCl 99%, sodium hydroxide NaOH 98%, anhydrous ethylene glycol C_2_H_6_O_2_ 99.8% and diiodomethane CH_2_I_2_ 99% were purchased from Sigma-Aldrich, (Saint Louis, Missouri) USA. Hydrochloric acid HCl 35% was obtained from Penta, Prague, Czech Republic. The reactants were used as received without any further purification.

Both sides of 5 × 5 × 0.1 cm PTFE foils were exposed to non-thermal air plasma by using the following plasma reactors: Pico (Diener electronic, Ebhausen, Germany) with a cylindrical chamber of 150-mm inner diameter Ø and 320 mm length operated at a frequency of 40 kHz, hereinafter (DC). Pico (Diener electronic, Ebhausen, Germany) Ø 150 mm and 320 mm length operated at a frequency of 13.56 MHz, referred to as (RF). The power inputs were 10, 20 and 50 W. The treatment durations were 0, 1, 2, 5, 10 and 20 min respectively, and the pressure in every experiment was 40 Pa. Once the treatment was completed, the specimens were withdrawn from the plasma reactor and immediately used for the next experiments. The foils were stored in a vacuum desiccator (MERCI S.R.O, Brno, Czech Republic) with stopcock, porcelain plate and cobalt chloride (CoCl_2_) (MERCI S.R.O, Brno, Czech Republic) indicating silica gel (MERCI S.R.O, Brno, Czech Republic).

### 2.2. Surface Wettability Assessment

Wettability of the samples was evaluated by contact angle measurement before and immediately after each modification. The sessile drop method was employed for this purpose on a Surface Energy Evaluation (SEE) system equipped with a CCD camera (Advex Instruments, Brno, Czech Republic). Deionized water, ethylene glycol and diiodomethane were used as testing liquids at 22 °C and 60% relative humidity. The droplets volume was set to 5 μL for all experiments. Every representative contact angle value was an average of 10 independent measurements. The substrate surface free energy was evaluated by using the acid-basic model.

### 2.3. Electrokinetic Analysis

**Figure 2:**
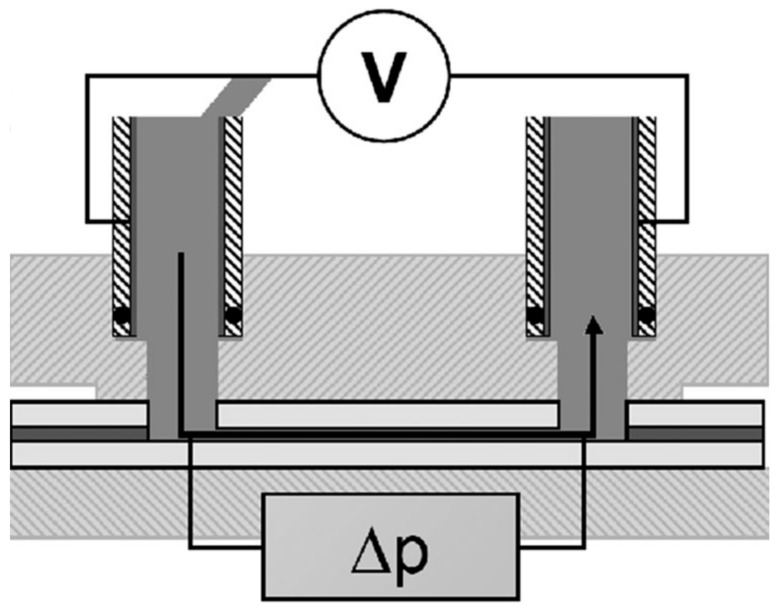
Schematic representation of a clamping cell for the determination of ζ-potential.

The ζ-potential of the sample surfaces was determined by using a SurPASS electrokinetic analyzer (Anton Paar GmbH, Graz, Austria) with a clamping rectangular measuring cell as the one shown in Figure 2. Streaming current and streaming potential measurement methods for flat solid surfaces were used (Anton Paar GmbH, Graz, Austria). The measurements were performed with 0.001M KCl as an electrolyte solution. The pH range was within 2–6 and adjusted by adding either NaOH 0.05M or HCl 0.05M.

### 2.4. Topographical Evaluation

The surface morphology was evaluated by using an Atomic Force Microscope (AFM) Solver PRO (NT-MDT, Moscow, Russia). The surfaces were analyzed with standard Si cantilever with a constant force of 10 N·m^−1^ and at resonance frequency of 170 kHz. In order to obtain a reliable result, the average surface roughness was obtained from different spots of the samples. The Scanning Electron Microscope (SEM) was carried out by using a Nova NanoSEM 450 (FEI, Brno, Czech Republic) with Schottky field emission electron source operated at acceleration voltage ranging from 200 V to 30 kV and low-vacuum SED (LVD) detector. A coating with a thin layer of gold was performed by a sputter coater SC 7640 (Quorum Technologies, Lewes, UK).

## 3. Results

### 3.1. Surface Energy Evaluation

**Figure 3:**
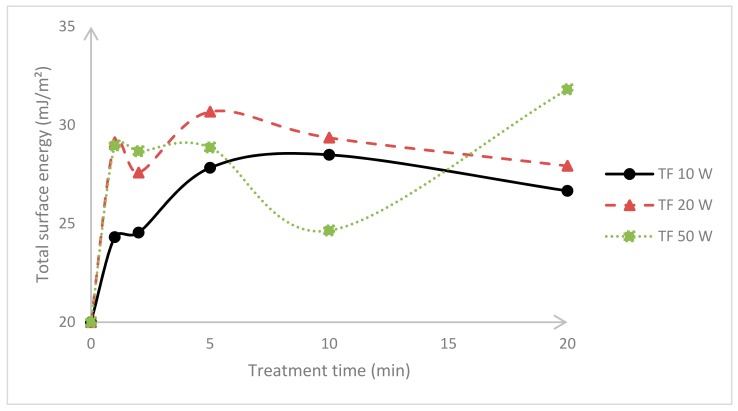
Surface energy of untreated and plasma-treated PTFE after using 40 kHz frequency (DC).

**Figure 4:**
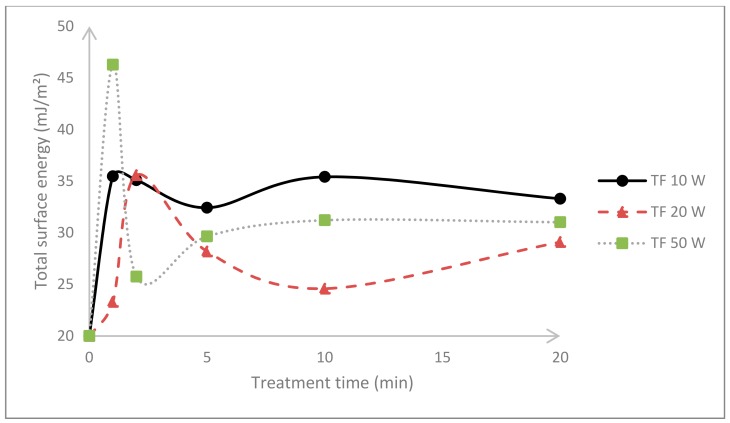
Comparison of the PTFE surface energy after radiofrequency (RF) plasma treatment with different plasma durations and power inputs.

In order to estimate the extent of plasma treatment at different treatment times and powers, the PTFE specimens were assessed by contact angle measurements. Figure 3 and Figure 4 depict the surface energy variation with respect to the plasma duration of either DC or RF plasma treatments.

With regard to the surface energy and surface wettability assessments, the contact angles of water, ethylene glycol and diiodomethane are listed in Table 1.

### 3.2. Surface Charge Appraisal

**Figure 5:**
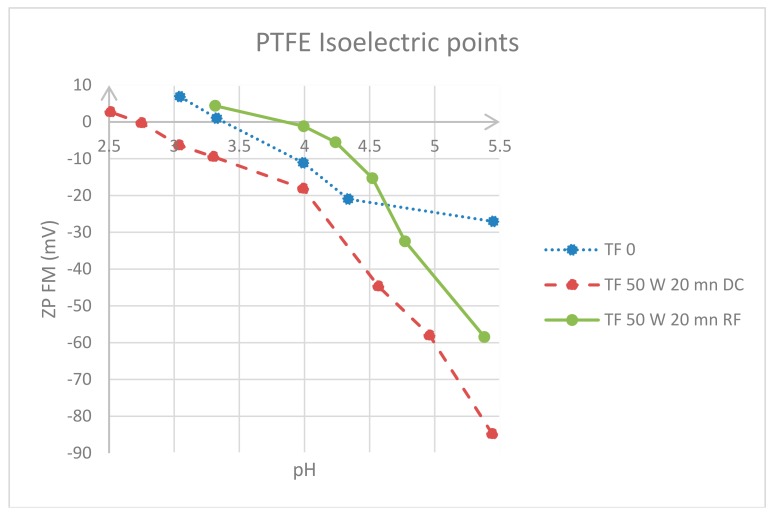
ζ-potential as a function of pH in aqueous solution of 0.001 M potassium chloride, and Isoelectric points (IEPs) of untreated and treated PTFE films.

The surface charge with respect to the plasma duration was appraised by electrokinetic analysis, where the ζ-potential is an indicator for charge formation at the solid-liquid interface and the surface charge is generated by the interaction of the solid surface with the electrolyte solution. Likewise, the isoelectric points (IEPs) are defined as the pH at which a substance has a net charge of zero, or at which it is at its minimum ionization. Figure 5 shows the trend of ζ-potential versus the pH and the isoelectric points of untreated PTFE as well as the treated sample after 20 min 50W DC and RF. For this experiment, only the longest durations and the highest power input were compared to untreated PTFE.

### 3.3. Topographical Assessment

**Figure 6:**
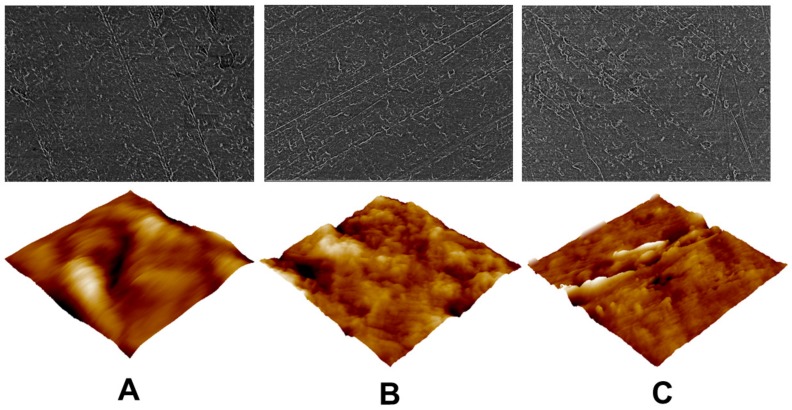
2D SEM and 3D AFM images of: (**A**) Untreated, (**B**) RF plasma treated, and (**C**) DC plasma treated PTFE samples.

**Figure 7:**
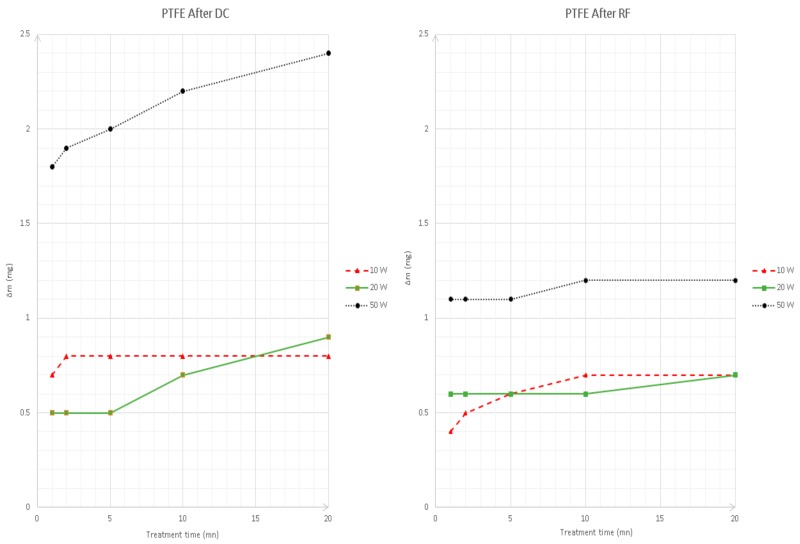
Effect of plasma treatment on the mass of the treated films.

Concerning the topographical patterning, the scanning electron microscopy (SEM) images along with the AFM ones of the examined specimens are presented in Figure 6. In addition, the connection between exposure time, mass change and by extension surface roughness is shown in Figure 7.

## 4. Discussion

Solid surfaces may be classified into two basic groups, hydrophilic (wettable with water and high surface energy) and hydrophobic (not wettable with water and low surface energy). The contact angles of the employed liquids on the studied surfaces diminished. For instance, the contact angle of deionized water decreased within the range 74–88°, which indeed indicates a surface wettability change. The typical water contact angle value of poly(tetrafluoroethylene) (PTFE) is ≥105 and its surface energy is typical 20 mJ/m^2^. The superhydrophobic character of PTFE plummeted after using air plasma treatment, demonstrating the capability of plasma treatment for surface modification [18,19,20]. Oxygen-containing (as air) plasmas increase the surface energy and introduce polar (O-containing) moieties. This phenomenon is a consequence of breaking bonds and free radicals formation that once the samples are withdrawn from the plasma reactor trigger the reaction between atmospheric oxygen and free radicals. The hydrophobic character of PTFE was altered making the specimens more hydrophilic after each treatment [21,22].

PTFE has a relatively low surface energy and its total surface energy increased regardless of the treatment. In the case of DC, the highest values correspond to the foils exposed to 20 min treatment with a power input of 50 W; nevertheless, there is no drastic surface energy change if shorter plasma durations are applied, and 1 min of treatment is enough for a cost-efficient surface modification [23,24,25]. This may be confirmed with the radiofrequency experiments, where it is evident that 1 min is sufficient as the further treatment or power input merely had an effect on surface energy. This phenomenon may be associated with chemical saturation after 1 min of plasma treatment. This is in agreement with previous studies, where short plasma duration is a rather cost-effective treatment. It should be noted that longer treatments might provoke thermal degradation that potentially damage the previously obtained surface attributes [26,27,28]. The treatment efficiency is intrinsically connected with experimental parameters of the plasma reactors; for example, pursuant to the plasma reactors’ supplier, the kHz machines are more robust, provide more uniform treatments, and are more efficient than any other commercial machines. The efficiency factor of 40-kHz frequency (DC) is 80%, whereas 13.56-MHz (RF) has the lowest efficiency factor of commercial plasma reactors, close to 50% [29,30].

As seen from the electrokinetic analysis, which was performed to evaluate the surface charge and to determine the isoelectric points of the studied sample, all the plots have negative values, and these negative charges increased after 20 min RF plasma treatment; conversely, the curve of 20 min RF shifted towards higher pH values. PTFE treated under DC holds the most negative ζ-potential values, which correspond to the highest surface charge. The untreated PTFE showed a value around pH 3.4, which coincides with the IEP found in literature [31]. The studied samples had negative charges indicating the presence of chemical groups which may be deprotonated. Poly(tetrafluoroethylene) is an inert and stable polymer, and its backbone may not be deprotonated; therefore, it may be assumed that new chemical groups were incorporated and strong monomer fragmentation occurred during the plasma treatment. The new surface-functionalities may act as electron donors (Lewis-base), which may explain why the curves drop to negative numbers [32].

With respect to the SEM images, both the untreated sample and the treated ones have wavy areas, and all studied sheets possessed scratches. These anomalies may come from the processing line or an inadequate storage. Nevertheless, the untreated film is relatively smoother and its morphology is more uniform than the treated ones. These alterations may be observed in more detail with the Atomic Force Microscopy (AFM) microphotographs, where the surface topography changes following the PTFE films’ exposure to air plasma treatment are more visible. The treated samples depict relatively rougher morphologies, with the sample treated under DC power being the roughest, with etched features and irregularly shaped textures compared with the untreated film. This may be substantiated in Figure 7, where the extent of plasma treatment was assessed with respect to the loss of weight. It may be noticed that the increase of Δm is proportional to the plasma duration and the power input. Hence, higher power input leads to greater weight loss. The foils treated for a longer period have rougher surfaces and underwent higher loss of mass. In fact, the generated pattern on the plasma treated samples stems from the competition between ablation and functionalization. It has been seen throughout this study that 1min under 40 kHz frequency is the most efficient in terms of surface modification, and this information is in agreement with previous studies, where, as it was mentioned above, the kHz machines are more robust, providing more uniform treatment, and are more efficient than other machines [33].

## 5. Conclusions

We conclude that the non-thermal plasma sources used in this work are suitable for the surface modification of poly(tetrafluoroethylene). The superhydrophobic character of PTFE is transformed after air plasma treatment. Different treatment times and powers were employed, and as far as this contribution is concerned, plasma treatment at 40 kHz is the most efficacious system and it is in agreement with previous studies. Short duration plasmas are cost-efficient methods to enhance surface properties without causing any negative impact on the treated samples. Surface charge and surface energy have been succinctly characterized by surface probe techniques. All the results demonstrate how the surface charge is gradually changed, and provide the moment that chemical saturation and thermal degradation begin. Surface energy increases with increasing treatment time. Physical and chemical changes are clearly manifested by the rise of surface charge after RF plasma treatment; likewise, the isoelectric point of treated PTFE after DC plasma treatment is lower than the untreated one. The microphotographs illustrate the surface morphology and the etching effects of the treatment, which are corroborated by the Δmass of the treated specimens. This contribution underlines the use of plasma treatment as a reliable tool for surface modification and functionalization.

## Figures and Tables

**Table 1:** Contact angles of PTFE samples after using DC and RF plasma treatments. The Lifshitz-Van der Waals/acid-base (LW/AB) theory was employed to obtain the total surface energy.

Sample		Contact Angle (°) After DC			Contact Angle (°) After RF		
Plasma Duration (min)	Power Input (W)	θ_w_	θ_e_	θ_d_	θ_w_	θ_e_	θ_d_
0	0	108.9	90.7	75.2	108.9	90.7	75.2
1	10	85.0	69.7	68.2	83.1	70.4	57.4
1	20	81.9	68.3	62.6	88.1	74.2	70.3
1	50	77.6	62.6	60.6	75.2	69.0	51.8
2	10	82.0	70.4	68.9	83.1	76.1	63.5
2	20	78.7	69.7	67.2	81.7	74.6	62.6
2	50	81.4	66.8	62.1	82.5	67.3	65.1
5	10	74.2	66.8	66.9	86.9	78.6	65.7
5	20	81.9	69.1	61.8	83.0	62.7	66.4
5	50	80.0	63.1	59.8	78.7	68.1	63.7
10	10	81.5	61.4	66.8	87.6	78.4	61.9
10	20	81.4	71.8	65.8	81.0	67.3	67.7
10	50	88.6	70.1	70.0	75.7	67.2	63.3
20	10	80.9	63.4	70.5	83.6	73.6	62.2
20	20	77.6	69.3	67.1	78.8	67.1	63.3
20	50	78.5	73.5	67.2	81.8	59.8	59.9
